# Fabricating a dielectrophoretic microfluidic device using 3D-printed moulds and silver conductive paint

**DOI:** 10.1038/s41598-023-36502-9

**Published:** 2023-06-12

**Authors:** Shayan Valijam, Daniel P. G. Nilsson, Dmitry Malyshev, Rasmus Öberg, Alireza Salehi, Magnus Andersson

**Affiliations:** 1 Faculty of Electrical Engineering, K. N. Toosi University of Technology, Tehran, 1631714191 Iran; 2 Department of Physics, Umeå University, 901 87 Umeå, Sweden; 3 Umeå Center for Microbial Research (UCMR), 901 87 Umeå, Sweden

**Keywords:** Lab-on-a-chip, Biomedical engineering, Characterization and analytical techniques

## Abstract

Dielectrophoresis is an electric field-based technique for moving neutral particles through a fluid. When used for particle separation, dielectrophoresis has many advantages compared to other methods, like providing label-free operation with greater control of the separation forces. In this paper, we design, build, and test a low-voltage dielectrophoretic device using a 3D printing approach. This lab-on-a-chip device fits on a microscope glass slide and incorporates microfluidic channels for particle separation. First, we use multiphysics simulations to evaluate the separation efficiency of the prospective device and guide the design process. Second, we fabricate the device in PDMS (polydimethylsiloxane) by using 3D-printed moulds that contain patterns of the channels and electrodes. The imprint of the electrodes is then filled with silver conductive paint, making a 9-pole comb electrode. Lastly, we evaluate the separation efficiency of our device by introducing a mixture of 3 μm and 10 μm polystyrene particles and tracking their progression. Our device is able to efficiently separate these particles when the electrodes are energized with ±12 V at 75 kHz. Overall, our method allows the fabrication of cheap and effective dielectrophoretic microfluidic devices using commercial off-the-shelf equipment.

## Introduction

**Figure 1:**
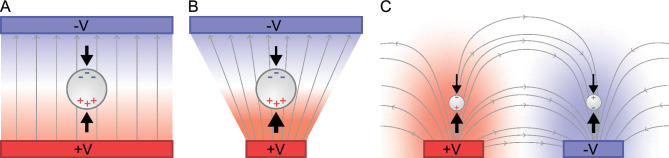
The principles of dielectrophoresis on a non-conductive neutral particle. Panel (**A**) shows that a neutral particle in a uniform AC field will have balanced forces and remain stationary. In panel (**B**), the AC field is non-uniform and this results in a net force on the particle that makes it move. If the frequency of the field is tuned so the particle moves toward the lower field intensity it is denoted as negative DEP. Panel (**C**) shows that when the electrodes are positioned next to each other (in a comb pattern), the electric field is stronger near the electrodes resulting in a repelling force, thus pushing the particles into the center of the microfluidic channel.

Microfluidic devices that can separate micro-particles have important applications in a variety of fields. These include medicine, chemical engineering, wastewater treatment, environmental assessment, forensic identification, and cell separation^1–4^. Separation of micron-sized particles can be done using different approaches; however, for both biological and non-biological particles, dielectrophoresis (DEP) has proven to be a powerful method. The technique offers label-free, rapid, and controllable manipulation, which has been shown to perform well in both low and for high-throughput applications^5,6^. Dielectrophoresis specifically refers to the interaction between a dielectric (non-conductive) particle, and a non-uniform, alternating electric (AC) field. Consequently, the DEP force depends both on the particle characteristics, such as size, morphology, and dielectric properties^7^; as well as the frequency of the electric field and how the electrodes are shaped and positioned, as shown in Fig. 1. If the electrodes are positioned next to each other, the non-uniform electric field near the electrodes results in a force deflecting the particles away from their path allowing for particle separation.

Effective particle separation requires an appropriate flow rate to transport the particles. This is often achieved using microfluidic channels, which allow for controlled and laminar flow conditions. Microfluidic devices, like those used for DEP, can be fabricated using various methods such as lithography, laser photoablation, hot embossing, and direct 3D printing^8,9^. Soft lithography is of special interest, as it is relatively cheap and easy to use. This technique uses bio-compatible polymers like polydimethylsiloxane (PDMS) to fabricate microfluidic structures around premade moulds. By printing these moulds using high-precision 3D printers, such as commercial stereolithography (SLA) printers, it is possible to produce moulds with spatial resolutions down to 5 μm^10–13^. This allows us to quickly produce small channels with a high degree of precision.

One common technique to generate wide electric fields for electrokinetic particle separation is by using planar comb electrodes. However, the fabrication process of these electrodes often requires expensive techniques like physical vapour deposition (PVD), micromachining, or inkjet printing^14–17^. Previous works have shown that it is possible to integrate the electrodes directly into the walls of a microfluidic channel using an Ag-PDMS mix; however making the nanoparticles distribute evenly throughout the PDMS has shown to be a challenge^18^. A more affordable and accessible technique for creating comb electrodes is directly applying them on the microfluidic channel. By creating a 3D-printed mould for the electrodes, it is possible to simply fill the mould with conductive material, such as silver conductive paint, and join the electrodes to the microfluidic channel.

In this work, we present a method for making DEP microfluidic devices for particle separation that is quick and does not need expensive equipment or a cleanroom. We design, optimize, and evaluate our device using finite element method (FEM) simulations. We then fabricate the device by using a 3D printer, PDMS moulding, and a conductive silver paint to create the microfluidic channels and comb electrodes. The electrodes are energized with AC voltage. Finally, we use high-speed imaging and particle tracking to verify and evaluate the separation of 3 μm and 10 μm polystyrene beads.

## Results and discussion

### Designing the electrodes and determining their electrical parameter using simulations

**Figure 2:**
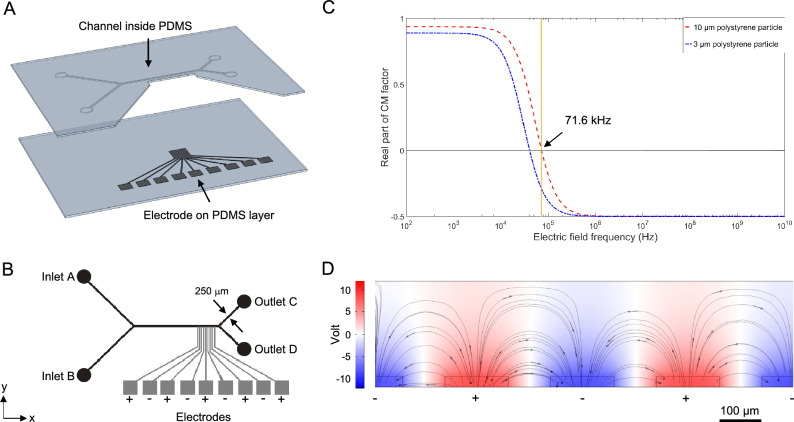
Panel (**A**) shows the design of the dielectrophoretic microfluidic device where the microfluidic channel (top layer) and electrodes (bottom layer) are placed on thin layers of PDMS. In panel (**B**), we show a compounded (top) view with all the features labeled. The electrodes extend into the channel and are routed back to connection pads that are energized with AC voltage. Panel (**C**) shows the CM factor plot. The real part of the CM factor is negative for both particle sizes for frequencies above 71.6 kHz (dotted line). Panel D shows a simulation of the relative electric fields in the comb electrode, when operated at 75 kHz.

To make a low-cost microfluidic device that can separate micro-sized particles using dielectrophoretic forces, we used a design-build-test approach. First, we designed the device using FEM simulations in COMSOL Multiphysics (v5.5, COMSOL AB). This allowed us to optimize the layout of the channels, electrodes, and electric parameters before constructing the physical device^19^. In our design, we considered that it should be possible to produce the channels and electrodes using a 3D printer for cheap and fast fabrication. The general design of the device is shown in Fig. 2A, with a detailed schematic in Fig. 2B showing the layout of the inlets, electrodes, and outlets. We made the main channel 250 μm wide, 35 μm high and 10 mm long, with approach channels extending out at 45$^{\circ }$ to the in- and outlet holes. These allow us to introduce and extract particles from the main channel. Along the side of the main channel, nine electrodes are positioned in a comb pattern. These are 150 μm wide, placed with a center-to-center distance of 250 μm and protruding 20 μm into the flow channel. The electrodes are made flush with the channel floor and extend to a depth of 35 μm into the bottom layer (electrode thickness). Both the top and bottom layers are made in PDMS (polydimethylsiloxane), which is a transparent, flexible and non-toxic silicone rubber. This compact design makes it possible to fit the dielectrophoretic microfluidic device on a microscope glass slide and observe the separation of biological samples in real-time.

We designed the device so that inlet A provides a controllable sheath flow through the main channel. This sheath flow focuses particles from inlet B towards the sidewall where the electrodes are positioned, enhancing the DEP-force felt by the particles. To generate an electric field between the electrodes, we connect them to +V and −V in an alternating pattern, see Fig. 2B. Our simulations suggest that particles are efficiently focused toward the electrodes using fluid rates of 70 μL/min and 14 μL/min for the sheath flow and particle flow, respectively. When particles enter the separation stage (region of the nine electrodes), they will be subjected to DEP forces in the y-direction, pushing them away from the electrodes. This repulsive DEP, where the particles move away from the strong electric field, is commonly referred to as negative DEP (nDEP). Depending on the size and electrical properties of the particles, the nDEP force will differ in magnitude, resulting in separation between the particle species. Our aim is to separate 3 μm and 10 μm polystyrene beads into outlets C and D. These particles are approximate size analogues to platelets and red blood cells, making them relevant to biological applications. We optimized this separation by tuning the layout of the device, the electrode voltage and the driving frequency. Since the driving frequency for the electrodes determines the nDEP force, we determined the Clausius-Mossotti (CM) factor by solving Eq. (3). The CM factor is a complex number that describes the polarizability of particles and its real part is shown in Fig. 2C for the polystyrene beads. A negative value results in a repulsive force on the particles and this occurs for both particle sizes at frequencies above 71.6 kHz. At these frequencies, it becomes possible to separate different-sized particles using nDEP, since the force is proportional to the cube of the particle radius, as seen in Eq. (2). Increases in the driving frequency will result in a larger nDEP force; however, high frequencies are known to cause air bubble formation, and as such, we use a driving frequency of 75 kHz in our experiments^18^.

**Figure 3:**
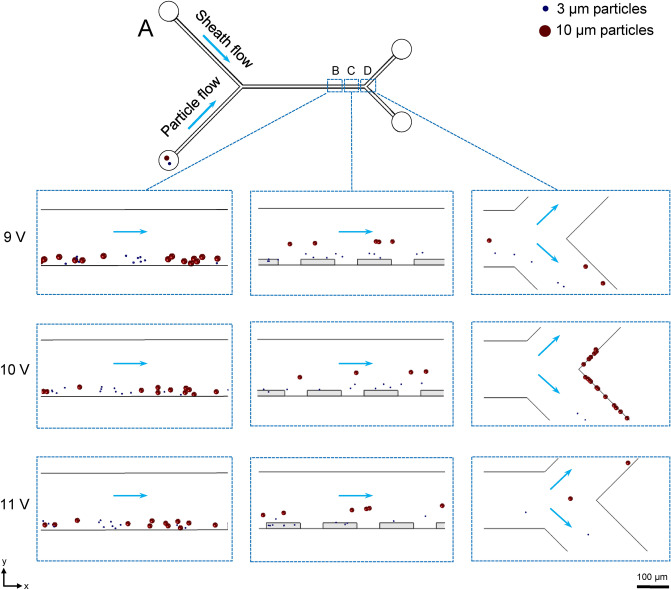
Numerical simulation to evaluate the voltage dependency on separation of 10 μm (red) and 3 μm (blue) particles. We test this for ±9, ±10, ±11 V and observe the particles in three key marked regions of our device: at the focusing stage (I), at the separation stage (II), at the separation junction (III). At 11 V, all particles are separated successfully according to their size.

Since we aim to make electrodes from silver conductive paint in our device, we produced prototype electrodes to evaluate their electrical properties and use these values in our simulation. We measured the conductivity of the electrodes to $5\times 10^{7}$ S/m, using a four-point probe. From the simulation, we calculated the electric field strength to be 0.017 V/μm in the center of the channel and 0.45 V/μm close to the electrodes, with the field distribution visualized in Fig. 2D. To find the lowest voltage that yields complete separation, we simulated a particle flow through the channel while energizing the electrodes at different voltages. In this simulation, we introduced 1 ml of buffer solution containing $\sim$4 000 randomly distributed particles ($\sim$2 000 of each size) at a constant flow rate. The properties of the particles and the buffer are listed in Table 1. During the simulation, we monitored the particle motion in three parts of the channel as seen in Fig. 3; before the separation stage (I), at the separation stage (II), and at the separation junction (III). From snapshots of these simulations for different applied voltages we found that ±9 V was insufficient to separate the two sizes (0 % of 10 μm particles flow through outlet C). By increasing the voltage to ±10 V, the particles are partially separated (42% of 10 μm particles flow through outlet C), and at ±11 V, particles are fully separated (100 % of 10 μm particles flow through outlet C). The particle motion in these simulations can be seen in the supporting movies S1–S3, respectively. We therefore conclude that energizing the electrodes at ±11 V (75 kHz) should be sufficient for the complete separation of 3 μm and 10 μm particles.

To further investigate how different flow rates influenced the purity and efficiency of the particle separation, Eqs. (9) and (10) respectively, we ran additional simulations. Both efficiency and purity were low at ±9 V, suggesting that such low voltage is unsuitable for our system. At ±11 V and ±13 V, both efficiency and purity are higher, but there was only a narrow range of flow rate values where both efficiency and purity are 100 %, as seen in Fig. S1. Increases in the electric field strength result in a higher separation efficiency; however, it also causes more Joule heating. Heating the solution will change its conductivity and permittivity, and excessive heating may harm biological cells^20^. To estimate how the temperature increases with applied voltage, we calculated the fluid temperature as

1 $$\begin{aligned} \Delta T\approx \frac{\sigma _{\text {m}}\times {V_{\text {rms}}}^2}{2K_{\text {m}}}, \end{aligned}$$

where $V_{\text {rms}}$ is the magnitude of the applied voltage, and using $\sigma _{\text {m}}$ = 100 mS/m and $K_{\text {m}}$ = 0.60 W/m K for the electrical and thermal conductivity, respectively^21^. At full separation (±11 V) we would have an increase of 10 K in the sample fluid, suggesting that voltages in this range will not significantly affect device performance or the particles.

### Fabricating the lab-on-a-chip device using 3D printed soft lithography

When building our lab-on-a-chip, we used a similar 3D printing and PDMS moulding approach as Ho et al.^22^. However, our device is made in two parts: the top layer containing the microfluidic channels, and the bottom layer containing the electrodes, as seen in Fig. 2A. To make moulds for these, the channels and electrodes from our simulation were inverted (protruding) and placed on a 1 mm thick backing, making the bottom of the mould. The moulds were prepared for the printer using a slicer software (Photon Workshop V2.1.29, Anycubic) and printed in high-resolution (50 μm) with a desktop SLA 3D printer (Photon Mono SE, Anycubic). Print settings had to be adjusted in the slicer software to ensure accurate dimensions, since settings like layer thickness and exposure times are dependent on both the printer and the resin. This calibration was first done using the coverslip method, as described in^23^, and then by printing and measuring open-source calibration models.

**Figure 4:**
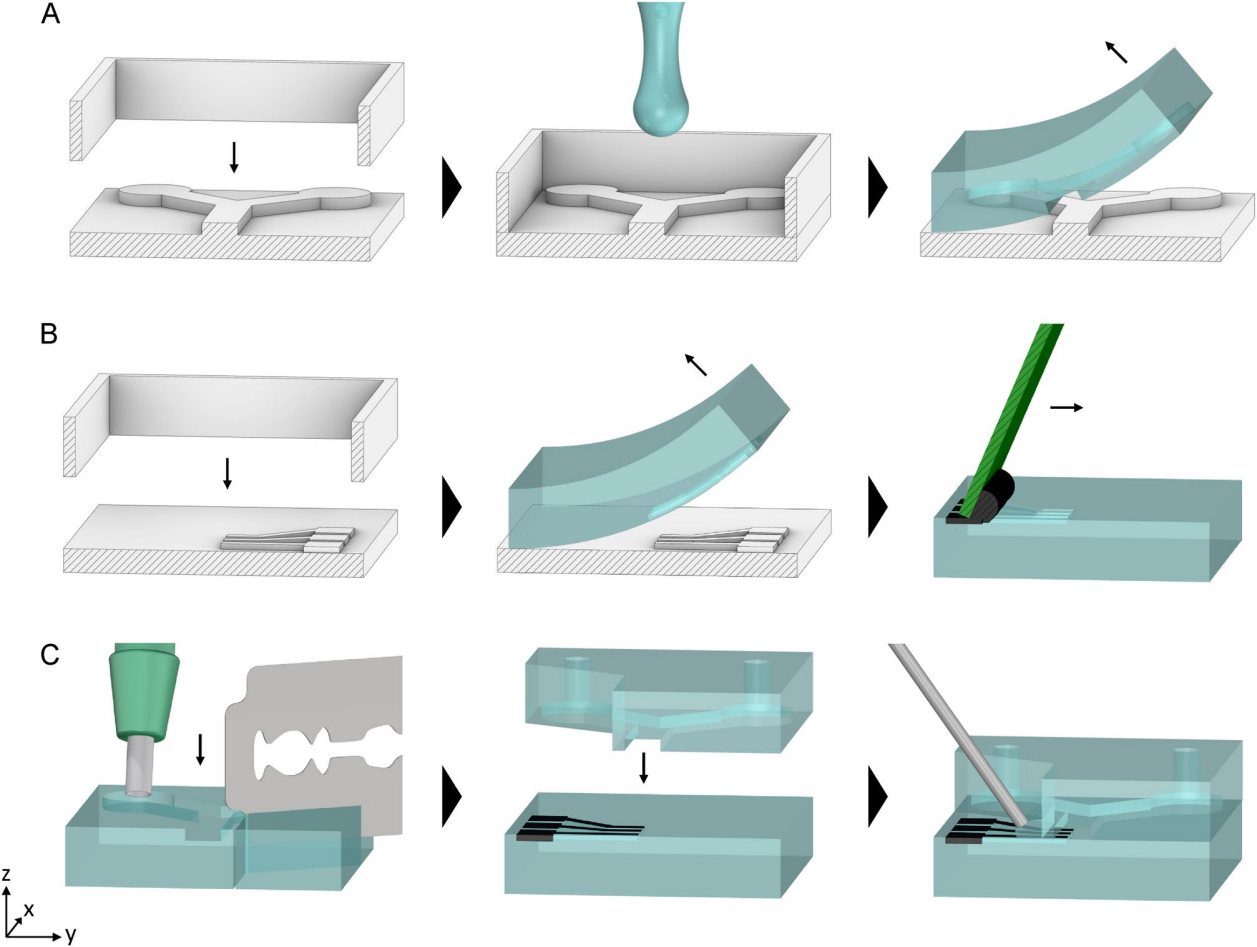
The main steps in the procedure of making a nDEP microfluidic device. For visualization purposes, the illustration is not to scale and cut in half along the yz-plane. In panel (**A**), we show how the top layer of the device is made. PDMS is poured into a 3D-printed mould (and its frame). Once cured, the PDMS is peeled off with an imprint of the flow channel. In panel (**B**), a similar procedure for the bottom layer is shown. The imprint for the electrodes is filled with silver conductive paint after the PDMS is cured. In panel (**C**), a biopsy punch is used to make openings in the channels before both halves are bonded together. To improve the bonding along the electrodes, we cut the corresponding part of the top layer away and added uncured PDMS at the seam using a syringe.

Before we could mould the PDMS layers, the printed parts had to be treated to make them inert, as failing to do so would inhibit the curing of the PDMS. This procedure consisted of three steps. First, cleaning the part in isopropanol for 3 min using a sonicator (Super RK 100, Bandelin Sonorex). Second, curing the part in a UV box (15 W at 400 ± 10 nm FWHM) for 2 h. Third, degassing the part in an oven at 70 $^\circ$C for 6 h. When the part was ready, it was clamped down (facing up) in a frame, filled with uncured PDMS to a thickness of 1 mm, and degassed in a vacuum desiccator. The PDMS (SYLGARD 184, Dow Corning) was prepared by mixing the elastomer base and the curing agent at a weight ratio of 10:1. Lastly, it was cured in an oven at 70 $^\circ$C for 1 h, before the PDMS layer with its imprint could be peeled off. This process was repeated for both parts of the device, as seen in Fig. 4A,B. The Young’s modulus of the PDMS were measured to 4.0[3] MPa ($95\%$ CI) using the compression gauge method^23^, as seen in Fig. S2.

In the top layer, through-holes were made for the in and outlets using a 1 mm biopsy punch (Integra 3331AA, Miltex). In the bottom layer, the imprints of the electrodes were filled with 3 μl silver conductive paint (SCP03B, Electrolube) and the excess was scraped off to make the electrodes flush with the channel floor. The paint consists of 45$\%$ silver particles, has a viscosity of 70 mPa•s (at 25 °C) and dries in $\sim$10 minutes. Both of the PDMS layers were then treated in O_2_ plasma for 1 min (0.5 mbar chamber pressure) using a plasma cleaner (Atto, Deiner Electronic), before being aligned and bonded together under an optical microscope. Finally, the device was placed on a hotplate for 5 min (at 90 °C) to improve the adhesion. However, it was found that some fluid could leak out where the electrodes bonded to the top layer. We therefore cut away the part of the top layer in contact with the electrodes and rebonded the two halves. Uncured PDMS was then poured on the exposed electrodes and cured to sealing the joint, as seen in Fig. 4C.

**Figure 5:**
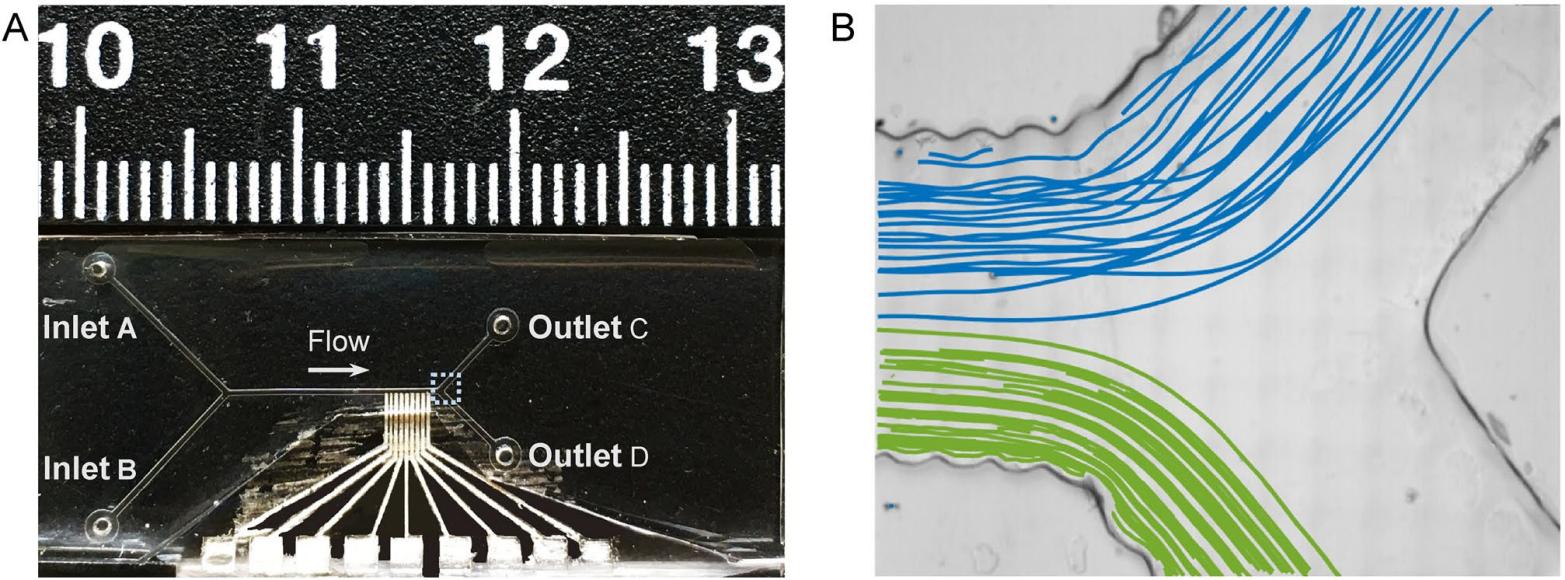
Panel A shows our microfluidic chip with the electrodes visible and a cm scale bar above. The separation junction is marked with a dashed box in Panel (**A**) and is shown in high resolution in Panel (**B**). Particle traces are shown for 3 μm (green) and 10 μm (blue) particles when energizing the electrodes at ±12 V. Particle traces before the separation stage and at the separation stage are shown in Fig. S3A,B.

Finally, nine copper wires were connected between the electrode pads and a signal generator (Model 8102, Topward), set to operate at ±11 V and 75 kHz. A photo of the finished device is shown in Fig. 5A. To allow fluid to flow through the device, we added tubing to the in and outlets, and connected the device to a syringe pump (VIT-FIT, LAMBDA Laboratory Instruments). The injection rates for inlets A and B were set to a continuous 70 and 14 μL/min, respectively, as suggested by the simulations. The channels were then washed with deionized water (for 2 min) ahead of use. Making our lab-on-a-chip requires a $200 SLA 3D printer and basic lab equipment. In terms of cost, manufacturing takes less than 2 h in labor time and $15 in materials. Thus, the fabrication is both accessible and low cost for laboratory users.

### Evaluating the separation efficiency using micro particles

To inlet B, we connected a syringe containing a phosphate-buffered saline (PBS) solution (0.1 S/m) and a mixture of 3 and 10 μm beads, as defined in section “Sample preparation”. The device was then placed in an optical microscope (Microphot-FX, Nikon Instruments) with a 20X objective (427958, Nikon Instruments) for tracking particles through the channel. Due to the high particle velocities, we mounted a high-speed CCD camera (MotionBLITZ EoSens Cube7, Mikrotron) and set it to record videos at 1696 × 1240 pixel resolution and 735 frames per second. Particle trajectories were analyzed from the video using the free tracking software, ToxTrac (v2.98)^24,25^. We first analysed region I (before the separation stage) to see if the flow ratio from the simulations would focus the particles to the side where the electrodes were positioned. Indeed, we observed that particles were focused closer to this side and that particles moved with the flow (not exposed to external forces), see Movie S4 and Figure S3A. Next, we analysed region II (separation stage) and region III (junction). Initially, when using the same voltage as in the simulation (±11 V), we observed that the particle separation was not complete ($purity < 1$). We therefore increased the voltage to ±12 V, at which full separation was achieved. Since the electric field is the cause of Joule heating, we recalculated the temperature increase using Eq. (1). At a separation voltage of ±12 V the total temperature increase is 12 K, which is only 2 K higher than at ±11 V, and still low enough to not affect the device or the sample significantly.

Running the device at ±12 V, we analysed the trajectories of the larger (10 μm) particles at the separation stage (electrodes 4 to 7) and found that these particles had a noticeable change in their y-directions, see Movie S5 and Fig. S3B. The gradient of the 10 μm particles was assessed to 0.042 ± 0.0013, by fitting a linear regression model to the trajectories. This implies that the 10 μm particles moved about 6.3 μm in the y-direction over a 150 μm distance (equal to the width of an electrode). After this, we monitored the separation junction and observed that particles were clearly separated by size, see Movie S6. To quantify this, we used ToxTrac and set the software to track particles according to size. Trajectories of about 30 particles from each group are shown in Fig. 5B, in which we have plotted 3 μm and 10 μm particles in green and blue, respectively.

### Characterizing the microfluidic channels and electrodes

When imaging the microfluidic channels, we noticed a wave-like pattern along the channel walls, see Fig. S3. These waves are caused by exposure bleeding (light leakage from the LCD pixels) during 3D printing and can be reduced by further tuning the exposure time of the printer or using light blocking additives for the resin. To ensure that these artifacts does not affect the particle separation efficiency, we remade the simulation with wave pattern along the walls, as seen in Fig. S4. The results shows no changes in the particle trajectories as compared to channels with straight walls. Rather, only subtle changes to the flow profile close to the channel walls were observed. This is similar to what we observed in the experiments, where only particles close to the walls follow a wave-like trajectory, but with no significantly impact to the particle separation efficiency.

**Figure 6:**
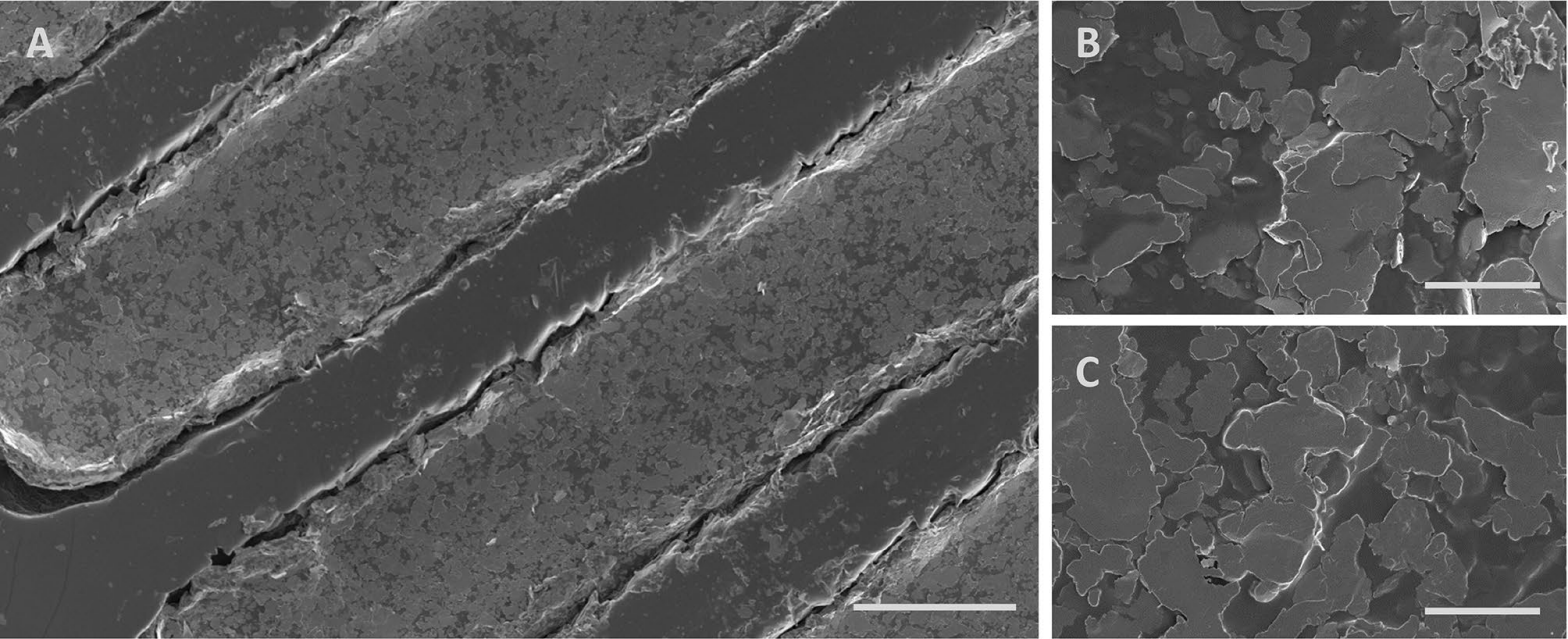
EM micrographs of the comb electrode after stress-testing with 15 V for 1 h, followed by immersion in a buffer solution and heating to 100 $^\circ$C. In panel (**A**), the electrodes are still showing an intact structure (scale bar is 100 μm). The microstructure of the electrodes at 5 000$\textrm{X}$ magnification is shown, before (**B**) and after (**C**) exposure, with no visual differences (scale bars are 10 μm).

To evaluate the robustness of the silver conductive paint electrodes, we tested their durability and observed their surface morphology. To test their durability, we energized the electrodes at a higher voltage than what was used during the experiments, ±15 V compared to ±12 V, at the same frequency of 75 kHz. The electrodes were also immersed in a buffer solution for 1 h and heated to 100 $^\circ$C. After these tests, we imaged the electrodes using SEM to evaluate their surface integrity, as shown in Fig. 6. We found no change to the surface morphology of the painted electrodes compared to controls, either on the macro-structure as seen in Fig. 6A, or on the microstructure as seen in Fig. 6B,C. Thus, we conclude that silver conductive paint electrodes are sufficiently durable for the purpose of our dielectrophoretic device. The silver paint electrodes could also be improved further by sintring, but for our purpose this was not required. Finally, since the electrodes does not have the same elasticity as the PDMS they can fracture from mechanical loads, so caution should be taken when handling the device.

## Conclusion

In this study, we developed a lab-on-a-chip device that utilizes dielectrophoresis to sort uncharged microparticles based on their size. We optimized our device for sorting 3 μm and 10 μm polystyrene beads by using FEM simulations to determine the driving frequency and voltage for the electrodes. We made the device from PDMS silicone rubber, using 3D-printed moulds that contain imprints of the channels and electrodes, and filled the electrodes with silver conductive paint. We evaluated the separation efficiency using particle tracking, showing our device could successfully separate 10 μm and 3 μm particles. Thus, our proposed method allows for the fabrication of cheap microfluidic DEP devices using common lab equipment. The versatility of the method can help in the production of more complex multi-component devices that can be used in various applications; such as, sorting cells, wastewater treatment, and separating bacterial spores from environmental samples for detection.

## Materials and methods

### Theoretical background

Dielectric (non-conductive) particles become polarized when placed in a non-uniform electric field. When polarised, these particles experience a force, the magnitude of which depends on the polarisability of the particle^26^. This is the dielectrophoresis (DEP) force, as shown in Fig. 1, and differences in this force can be used to redirect and sort particles. For homogeneous spherical particles, the DEP force is given by

2 $$\begin{aligned} F_{\text {DEP}}=2\pi r^3\varepsilon _{\text {0}}\varepsilon _{\text {m}}\text { Re}[CM(f)]\nabla \cdot\left|\vec {E}\right|^2, \end{aligned}$$

where $r$ is the radius of the particle, while $\varepsilon _{\text {m}}$ and $\varepsilon _{\text {0}}$ are the permittivity of the suspension medium and vacuum, respectively. The gradient of the electric field is given by $\nabla \cdot\left|\vec {E}\right|^2$. Finally, Re$[CM(f)]$ represents is the real part of the Clausius-Mossotti $(CM)$ factor. The $CM$ factor can vary between $-0.5$ and 1.0 and is dependent on the particle’s complex permittivity and thus the operating frequency *f* of the applied electric field. This factor also determines what direction the particle will move^27^. If $CM$ is positive, the DEP force is directed towards the stronger electric field, and is referred to as pDEP. If the $CM$ factor is negative, the force is directed away from the stronger electric field, and is called nDEP^28^. The device we use in this study works with nDEP, as shown in Fig. 2C. The $CM$ factor is quantified as

3 $$\begin{aligned} CM=\frac{\varepsilon _{\text {p}}^*-\varepsilon _{\text {m}}^*}{\varepsilon _{\text {p}}^*+2\varepsilon _{\text {m}}^*}. \end{aligned}$$

where the complex permittivity of the particle and medium are given by $\varepsilon _{\text {p}}^*$ and $\varepsilon _{\text {m}}^*$, respectively. Thus the $CM$ factor value can be between -0.5 ($\varepsilon _{\text {p}}^*$ $<<$ $\varepsilon _{\text {m}}^*$) and 1 ($\varepsilon _{\text {p}}^*$ $>>$ $\varepsilon _{\text {m}}^*$), as mentionned previously. These two parameters are defined as

4 $$\begin{aligned} \left\{ \begin{array}{lll} \varepsilon _{\text {p}}^*= \varepsilon _{\text {p}}-j\frac{\sigma _{\text {p}}}{w}, \\ \varepsilon _{\text {m}}^*= \varepsilon _{\text {m}}-j\frac{\sigma _{\text {m}}}{w}, \\ \end{array} \right. \end{aligned}$$

where the conductivity of the particle and medium are $\sigma _{\text {p}}$ and $\sigma _{\text {m}}$, respectively. Further, $w=2\pi f$ is the angular frequency and $j = \sqrt{-1}$. The other major force affecting the movement of the suspended particles in microfluidic devices, is the hydrodynamic drag force. Hydrodynamic drag force for spherical particles in laminar flow is calculated from Stokes’ law

5 $$\begin{aligned} F_{\text {Drag}}=6\pi r_{\text {ext}} \eta (\vec {u}-\vec {u}_\text {p}), \end{aligned}$$

where $r_{\text {ext}}$ is the external radius of the particle, $\eta$ is the viscosity of the medium, $\vec {u}$ and $\vec {u}_{\text {p}}$ are the flow velocity and the particle velocity, respectively. We defined the physical and electrical properties of the particles and the medium as described in the literature^29,30^, and the parameters used in our simulations are listed in Table 1.

### Numerical simulation

We simulated the device using a three dimensional model in COMSOL Multiphysics (v5.5, COMSOL AB). We used the laminar flow module to solve the Navier-Stokes equation and obtain the flow field. The AC/DC module was used to simulate non-uniform electric field by solving Laplace’s equation. The fluid (water) was assumed incompressible, and the flow was assumed to be laminar and steady. Both these are reasonable assumptions for flows at these low Reynolds numbers. A non-slip boundary condition was applied to the channels, and the pressure at all inlets and outlets was kept zero. A constant flow was assumed for the inlets, with the injection flow rate for inlet A and B set to 70 μL/min and 14 μL/min, respectively. Newton’s second law was employed to solve the particle trajectory, given by

6 $$\begin{aligned} \left\{ \begin{array}{lll} \nabla {p}+\eta \nabla ^{2}{\vec {u}}+\vec {F}_{\text {e}}=0, \\ \nabla ^{2}{u}=0, \\ \nabla \vec {u}=0, \\ \end{array} \right. \end{aligned}$$

where $P$ and $\vec {F}_{\text {e}}$ are the pressure and applied forces, respectively. The properties of the suspension medium were set to those of water, with density 1000 kg/m$^3$, dynamic viscosity 1.0$\times 10^{-3}$ Pa s, and relative permittivity 80^31^. The Laplace equation was solved to investigate applied electrical potential

7 $$\begin{aligned} \nabla ^{2}{\varphi }=0, \end{aligned}$$

where $\varphi$ is the electric potential applied to the electrodes, while all other boundaries were considered insulating. By solving Eqs. (2) and (4), the position of the particles could be calculated using

8 $$\begin{aligned} {\vec {F}_{\text {DEP}}} +{\vec {F}_{\text {drag}}}=m\dfrac{d\vec {u}_{\text {p}}}{dx} \end{aligned}$$

where $m$ and $t$ are the mass of the particle and time, respectively. Our simulation model consisted of 38 886 mesh elements in total.

**Table 1:** Physical and dielectric properties of the particles and suspension medium (buffer).

Property	Particle		Buffer
	10 μm	3 μm	
Conductivity (S/m)	2.5e−2	4.5e−2	0.1
Relative permittivity	5.0$\times \varepsilon _{\text {0}}$	2.56$\times \varepsilon _{\text {0}}$	80$\times \varepsilon _{\text {0}}$
Dynamic viscosity (Pa$\cdot$s)	–	–	1.0e−3
Density (kg/m$^3$)	–	–	1.0e+3

### Sample preparation

To test the performance of the device, we prepared a mixture of 3 ± 0.09 μm (C37484, Invitrogen CML Latex, Thermo Fisher Scientific) and 10 ± 1.0 μm (C37259, Invitrogen CML Latex, Thermo Fisher Scientific) latex polystyrene beads. The stock concentration for 10 μm and 3 μm particles was 4.1 g/mL and 4.3 g/mL, respectively, and both stocks were diluted 3000 times with deionized water to achieve appropriate concentrations. To make the medium better suited for nDEP, we modified the electrical conductivity of the particle and sheath flow medium to 0.1 S/m, by adding 1X phosphate-buffered saline (PBS) solution at a volume ratio of 1:100.

### Particle tracking

To track the particles in our microchannel, we used the free image tracking software called ToxTrac (v2.98)^24^. Videos were first preprocessed in UMUTracker^32^ to remove the background and binarize the videos. The videos were loaded in ToxTrac and we defined the region that we wanted to track as an arena. In ToxTrac, we could set the tracking algorithm to track particles depending on their size. Since some particles had low contrast and were hard to track between frames, we used the multitracking ID-algorithm to have higher stability in the trajectories^25^. Despite this, some trajectories were broken, but this did not affect the final results since we could merge broken tracks or just identify particles based on their sizes when exiting either outlet A or B.

To calculate the particles’ speed in the y-direction at the separation region, we fitted a linear regression model to individual trajectories. We multiplied this number with the pixel to μm conversion factor (2.5 pixel/μm), defined by the sensor’s pixel size (8 μm) and microscope magnification (20X).

### SEM imaging

We used SEM to image the silver conductive paint electrodes after different treatments. To prevent charge buildup on the PDMS surface, we coated the sample with a 5 nm thick layer of platinum using a sputter coater (Q150T-ES, Quorum Technologies Ltd). We then imaged the electrodes using a scanning electron microscope (Merlin FESEM, Carl Zeiss), with its InLens imaging mode at a magnifications of 100–50000$\textrm{X}$.

### Definitions of purity and separation efficiency

We tested the particle separation system at different voltages and flow rates to see how well it captured 10 μm particles (purity) within a background of other particles and how many were collected compared to the total amount of infused 10 μm particles (efficiency). Purity and efficiency are thereby defined as,

9 $$\begin{aligned} {Purity=\dfrac{Number\,\, of\,\, 10 \mu m\,\, particles\,\, (Outlet)}{Total\,\, number\,\, of\,\, particles\,\, (Outlet)}}, \end{aligned}$$

10 $$\begin{aligned} {Efficiency=\dfrac{Number\,\, of\,\, 10 \mu m\,\, particles\,\, (Outlet)}{Number\,\, of\,\, 10 \mu m\,\, particles\,\,(Inlet)}}. \end{aligned}$$

## Supplementary Information

Supplementary Information 1.

Supplementary Information 2.

Supplementary Information 3.

Supplementary Information 4.

Supplementary Information 5.

Supplementary Information 6.

Supplementary Information 7.

## Data Availability

The datasets generated during the current study are available from the corresponding author on reasonable request.
