# Supplementary material for: Fabricating a dielectrophoretic microfluidic device using 3D-printed moulds and silver conductive paint
Source: Sci Rep. 2023 Jun 12;13:9560. doi: 10.1038/s41598-023-36502-9 (PMC10260938; doi:10.1038/s41598-023-36502-9)
Supplement: Supplementary file 7 — Supplementary Information 7. [file 41598_2023_36502_MOESM7_ESM.pdf]

Fabricating a dielectrophoretic microfluidic device using 3D printed molds and silver conductive paint

Shayan Valijam[1,2], Daniel P.G. Nilsson, Dmitry Malyshev[2], Rasmus Öberg[2], Alireza Salehi[1], Magnus Andersson[2,3,*]

[1]Faculty of Electrical Engineering, K. N. Toosi University of Technology, Tehran, 1631714191, Iran

[2]Department of Physics, Umeå University, Umeå, 901 87, Sweden

[3]Umeå Center for Microbial Research (UCMR), Umeå, 901 87, Sweden

[*]Corresponding author: magnus.andersson@umu.se

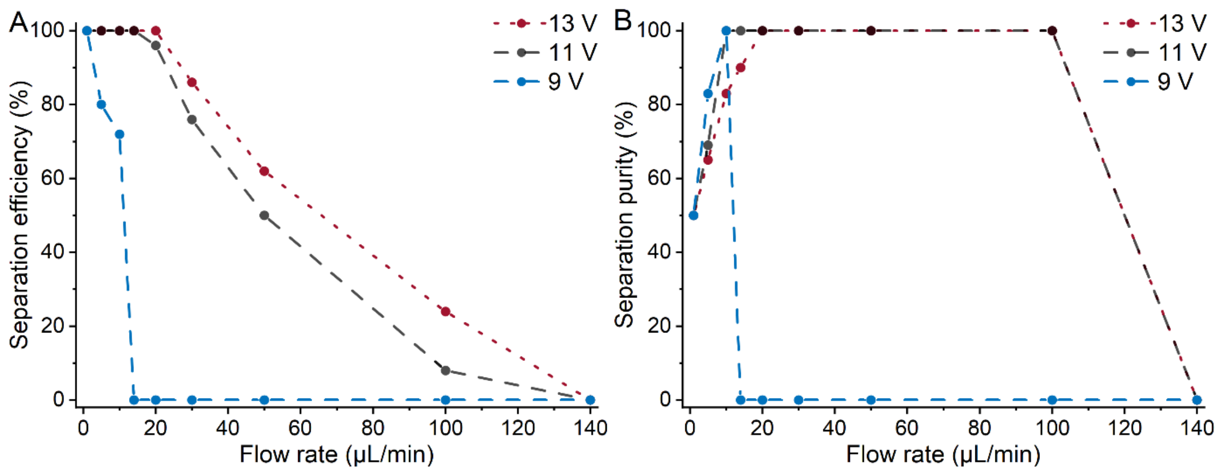

Figure S1. Simulated separation efficiency (A) and purity (B) of 10 µm particles in the microfluidic chip at different voltages and fluid flow rates. The separation efficiency is the amount of collected 10 µm particles over the total amount of 10 µm particles infused through the device. The purity describes the ability of the device to separate 10 µm particles from the 3 µm particles.

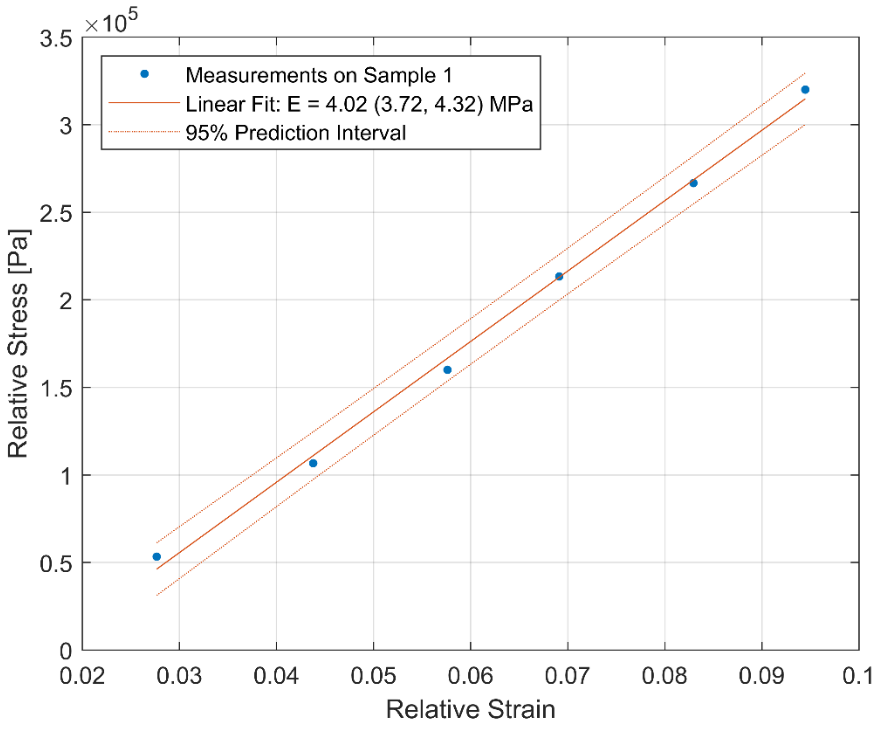

Figure S2. Measuring the Young's modulus as the slope between stress and strain, using the compression gauge method [1]. The measurement was performed on the PDMS layers containing the channel and electrodes, and after a steady state was reached.

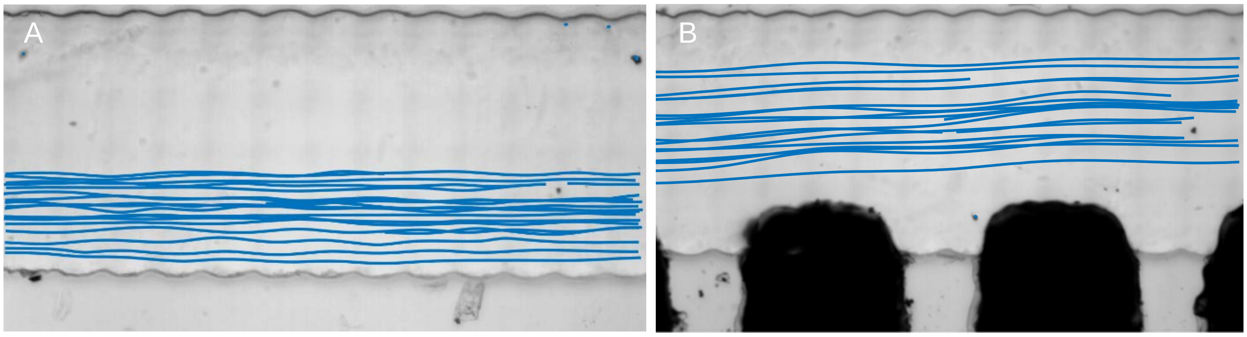

Figure S3. Examples of 10 µm particle trajectories A) before the electrodes and B) at the electrodes.

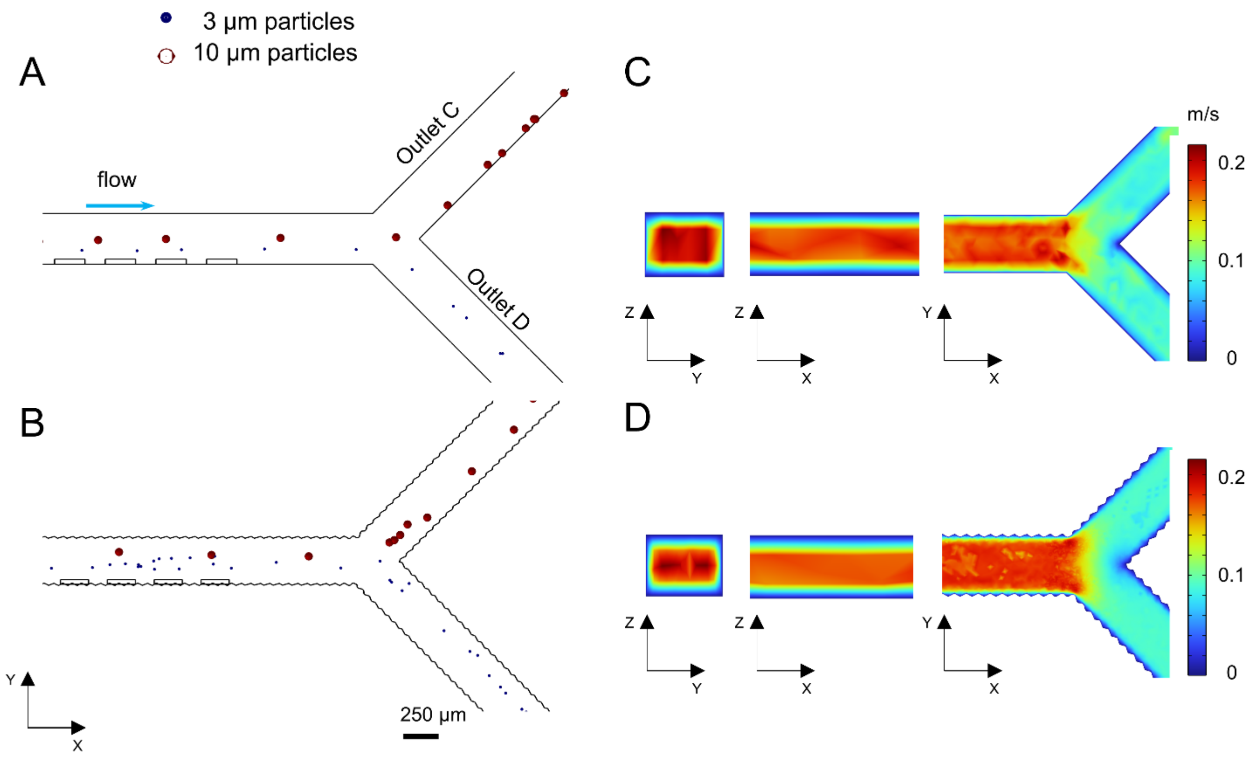

Figure S4. Still image from the simulation of the separation of particles with V ±11 V at 75 kHz. The wave like pattern does not interfere in the ability to separate the particles (A-B). The flow velocities inside the channel (C-D) are also similar.

Supporting movies

Movie S1 – Simulation with electrodes energized with 9 ±V

Movie S2 – Simulation with electrodes energized with 10 ±V

Movie S3 – Simulation with electrodes energized with 11 ±V

Movie S4 – Video recording before the electrodes

Movie S5 – Video recording at the electrodes

Movie S6 – Video recording of the junction
